# Three unconventional maxims in the natural selection of cancer cells: Generation of induced tumor-suppressing cells (iTSCs)

**DOI:** 10.7150/ijbs.79155

**Published:** 2023-02-27

**Authors:** Kexin Li, Qingji Huo, Bai-Yan Li, Hiroki Yokota

**Affiliations:** 1 Department of Pharmacology, School of Pharmacy, Harbin Medical University, Harbin 150081, China; 2 Department of Biomedical Engineering, Indiana University Purdue University Indianapolis, Indianapolis, IN 46202, USA; 3 Indiana Center for Musculoskeletal Health, Indiana University School of Medicine, Indianapolis, IN 46202, USA; 4 Simon Comprehensive Cancer Center, Indiana University School of Medicine, Indianapolis, IN 46202, USA

**Keywords:** natural selection, tumor proteome, iTSCs, conditioned medium, protein cocktail

## Abstract

Induced tumor-suppressing cells (iTSCs) can be generated from cancer and non-cancer cells. Here, three paradoxical maxims for the action of iTSCs are reviewed: the secretion of tumor-suppressing proteins, their role as a “double-edged” sword, and the elimination of lesser-fit cancer cells. “Super-fit” cancer cells secrete an array of proteins, most of which contribute to enhancing their growth and removing “lesser-fit” cancer cells. These maxims explain the potential dilemma with therapeutic agents since the inhibitory agents tend to promote the synthesis of tumor-promoting proteins. The maxims suggest the possibility of a novel treatment option using cancer-guided evolutionary-fit iTSCs.

## Natural Selection and Therapeutic Strategy

Cell competition eliminates cells that are viable but less fit than the surrounding cells 1. Therapeutically, cell competition could play a beneficial role by removing damaged cells and eliminating cells carrying oncogenic mutations (reviewed by 2, 3). In most cancer treatments, however, cell competition is a difficult problem to address since it promotes the expansion of oncogenic clones and the development of resistance to treatments 4. In their review, Parker *et al*. suggest that cancer cells acquire a super competent phenotype by steadily accumulating genomic aberrations that promote the death of surrounding healthy cells to initiate and promote oncogenesis 5. As asked by Bowling *et al*. 1, a central question herein is whether it is possible to harness cell competition for the benefit of treating primary and advanced metastatic cancer. If “yes”, what therapeutic strategy should be developed?

By citing Dobzhansky's remark on evolution, “nothing in biology makes sense except in the light of evolution,” Greaves stated that the therapeutic resistance of advanced cancers is a consequence of a complex, dynamic, and adaptive environment, underpinned by genetic diversity and epigenetic plasticity 6. He proposed a three-step strategy to control cancer, including (1) stopping it before it gets started by avoiding cigarettes and UV, and maintaining healthy diets and physical activities, (2) detecting it early and removing it when localized by a frontline therapy such as radiation and surgery, and (3) taming it with drug combinations including immunotherapy when cancer has already progressed to an advanced or metastatic state 7. As a whole, the strategy indicates the necessity of intervening at all three phases in cancer's evolutionary trajectory, as well as highlighting the difficulty in eliminating cancers at a late stage.

By viewing evolving cancer cells in another natural selection framework, Reed *et al*. proposed an extinction and adaptation strategy 8. In the extinction stage, the first strike is the application of large-scale and high-impact cytotoxic drugs, which mimic a catastrophic extinction in the evolution of life. If not eliminated, however, the continued use of the first-strike therapy is evolutionarily unwise because the remaining cells acquire resistance. The second strike should therefore take different approaches to push cancer cells below their extinction threshold. When extinction with the first and second strikes becomes impossible, adaptive therapy, which is analogous to integrated pest management, is preferred. In integrated management, therapy is halted while cancer cells are declining and reapplied upon their re-emergence. The goal of adaptive therapy is to limit cancer progression while retaining the sensitivity of cancer cells to therapeutic agents.

The above two strategies, both inspired by the Darwinian theory of natural selection, commonly propose the rapid elimination of primary cancer cells without allowing them to develop an evolutionary rescue in the initial treatment phase. If elimination is not possible, the later adaptive phase aims to restrain continued growth and drug resistance by permitting a portion of treatment-sensitive cancer cells to survive 9. In their recent review article, Gatenby and Brown described a new concept of integrating evolutionary dynamics into cancer therapy by focusing on the eco-evolutionary dynamics of treatment-resistant cancer populations 10. Notably, the evolutionary legacies of cancer cells, which can generate an astronomical number of subclones, are often superior to conventional therapy, which does not alter the combination of drugs at the same scale and pace as evolving cancer cells. An intriguing question is whether we can learn from their “survival-of-the-fittest” principle and develop a counteractive therapeutic strategy.

During natural selection, super-fit cancer cells may have at least two tactics to eliminate neighboring cancer cells. One tactic is to enhance their own metabolism, proliferation, and migration, while a second tactic is to kill competitors or weaken their cellular capabilities. If evolutionary machinery is economically implemented, these two tactics might be achieved using the same regulatory block, for instance employing context-dependent moonlighting proteins. A proposed hypothesis is that super-fit cancer cells achieve these two tactics using a group of proteins that act as tumorigenic agents intracellularly and serve as anti-tumorigenic agents to other cancer cells extracellularly. Here, we hope to “learn from cancer” to understand how cancer cells become super-fit and eliminate neighboring cancer cells.

## Three Unconventional Maxims

According to Bowling *et al*. 1, no clear consensus has been established on any feature that determines “competitive cell fitness”, whereas aggressive cancer cells tend to grow rapidly. Focusing on cell proliferation and tumorigenic signaling, we aimed to build super-fit cells, named induced tumor-suppressing cells (iTSCs), which could eliminate less-fit neighboring tumor cells by secreting tumor-suppressing proteins. The Cancer Genome Atlas (TCGA) provided the landscape in tumorigenic signaling pathways, and the research group led by Schultz investigated the mechanisms and patterns of somatic alterations in 10 canonical pathways 11: cell cycle, Hippo, Myc, Notch, Nrf2, PI3K/Akt, RTK-RAS, TGFβ, p53, and β-catenin/Wnt. So far, we have reported the successful generation of iTSCs from ~10 cell lines of breast cancer, prostate cancer, and pancreatic cancer, as well as non-cancer cells such as mesenchymal stem cells (MSCs), osteoblasts, pre-osteoclasts (macrophages), osteocytes, peripheral blood mononuclear cells (PBMCs), T lymphocytes, and monocytes. Their generation was achieved by overexpressing cMyc, β-catenin, Lrp5 (Wnt co-receptor), Snail (EMT inducer), Oct4 (one of the four transcription factors to produce induced pluripotent stem cells), and activating TGFβ, PI3K/Akt, and Wnt pathways by biological and chemical agents 12-20. According to the materials and methods section of the Li *et al*. study 16, transient overexpression of the above genes was conducted using plasmid transfection. Approximately 2 × 10^5^ host cells were grown in a 60-mm plate and the transfection of genes such as cMyc, β-catenin, Lrp5, etc. was conducted using a Lipofectamine 3000 reagent (L300015, Thermo Fisher Scientific, Waltham, MA, USA). The DNA solution was incubated for 10-15 min at room temperature, and the transfection was performed overnight. Based on the available studies 13-21, we present three unconventional maxims of cancer cells:

- Proliferating tumor and non-tumor cells remove neighboring tumor cells by secreting tumor-suppressing proteins.
- Some secreted tumor-suppressing proteins are oncogenic inside the cell.
- Secreted tumor-suppressing proteins preferentially kill tumor cells more than non-tumor cells.

**Tumor-suppressing proteins**: In the review article by Madden *et al*. 22, the tumor cell secretomes, an array of tumorigenic factors released by tumor cells, are introduced as an emerging mechanism of chemoresistance. Cancer cells release tumorigenic factors to prevent chemotherapy-dependent cytotoxicity. Chemotherapy exposure can change the types and abundance of components in the secretomes. We may also consider the reciprocal logic: if “chemo agent-treated cancer cells produce tumor-promoting proteins,” then do “growth-promoting agent-treated cancer cells produce tumor-suppressing proteins”? Therefore, a mirror-image question is whether the secretomes become anti-tumorigenic when tumor cells are exposed to a growth-stimulatory agent.

The action of iTSCs can be viewed from cell competitions that are observed during Drosophila organogenesis as well as mouse embryogenesis 23-25. According to Johnston *et al*.l when a group of Drosophila cells expressed a higher level of dmyc (a homolog of c-Myc) than their neighbors, they outcompeted neighboring cells and even killed wild-type cells further away 23. Furthermore, when dmyc-overexpressing cells were co-cultured with wild-type cells, the resulting conditioned medium was reported to induce cell death when incubated with wild-type cells 24. A murine embryo study also demonstrated that a mosaic imbalance of Myc expression provokes the expansion of cells with higher Myc levels through the apoptotic elimination of cells with lower levels 25. We observed in iTSC studies that when an oncogene cMyc is overexpressed in several cancer cell lines, all of their conditioned media (CM) present tumor-suppressive capabilities 16.

Besides c-Myc, Kirsten rat sarcoma viral oncogene homolog (Kras), a small GTPase transductor protein, is the other well-known oncogene with a high mutation rate among all cancers, including pancreatic ductal adenocarcinoma, nonsmall-cell lung cancer, and colorectal cancer (reviewed in 26, 27). Surprisingly, however, Zhang *et al.* reported that mice with a heterozygous Kras deficiency were highly susceptible to the chemical induction of lung tumors when compared to wild-type mice 28. Furthermore, wild-type Kras inhibited colony formation and tumor development by a mouse lung tumor cell line containing an activated Kras allele. This study indicates the possibility of generating iTSCs by the overexpression of Kras.

The efficacy of tumor-suppressive CM differs depending on the host cells such as breast, prostate, and pancreatic cancer cells, bone marrow-derived MSCs, PBMCs, and lymphocytes, as well as the pathways to be engineered. For instance, the activation of Wnt signaling is effective for most cancer cells and MSCs, but not for PBMCs and lymphocytes. These blood cells can be converted into iTSCs by the activation of PKA signaling 29. Little is known about the compatibility of host iTSCs to the pathways to be modulated. The anti-tumor ability is given collectively by many proteins in CM, and preclinical studies using mouse models revealed that the systemic administration of iTSC CM suppressed the growth of mammary tumors and blocked the progression of metastasized cancer cells in the bone and brain 12, 13, 15, 16. One of the unexpected findings of tumor proteomes is that many tumor-suppressing proteins, which are enriched in the CM, have been known as tumor-promoting proteins. Their location-dependent double-edged activity may not be always consistent with the inhibition of oncogenic targets.

While tumor-suppressing proteins in iTSC CM inhibit the progression of tumor cells by elevating cleaved caspase 3 which is a key apoptosis-inducing caspase 30, the exact extrinsic and intrinsic apoptosis pathways have not been clarified. In our studies 16, 18, the interaction of enolase 1 (ENO1), to CD44 was shown to lead to apoptosis. ENO1 is a tumor-suppressing protein that is enriched in iTSC CM. Since the inhibition of CD44 is reported to induce apoptosis and inflammation in skeletal tissues 31, the interactions of atypical tumor-suppressing proteins with cell-surface proteins such as CD44 may trigger apoptosis.

**Figure 1:**
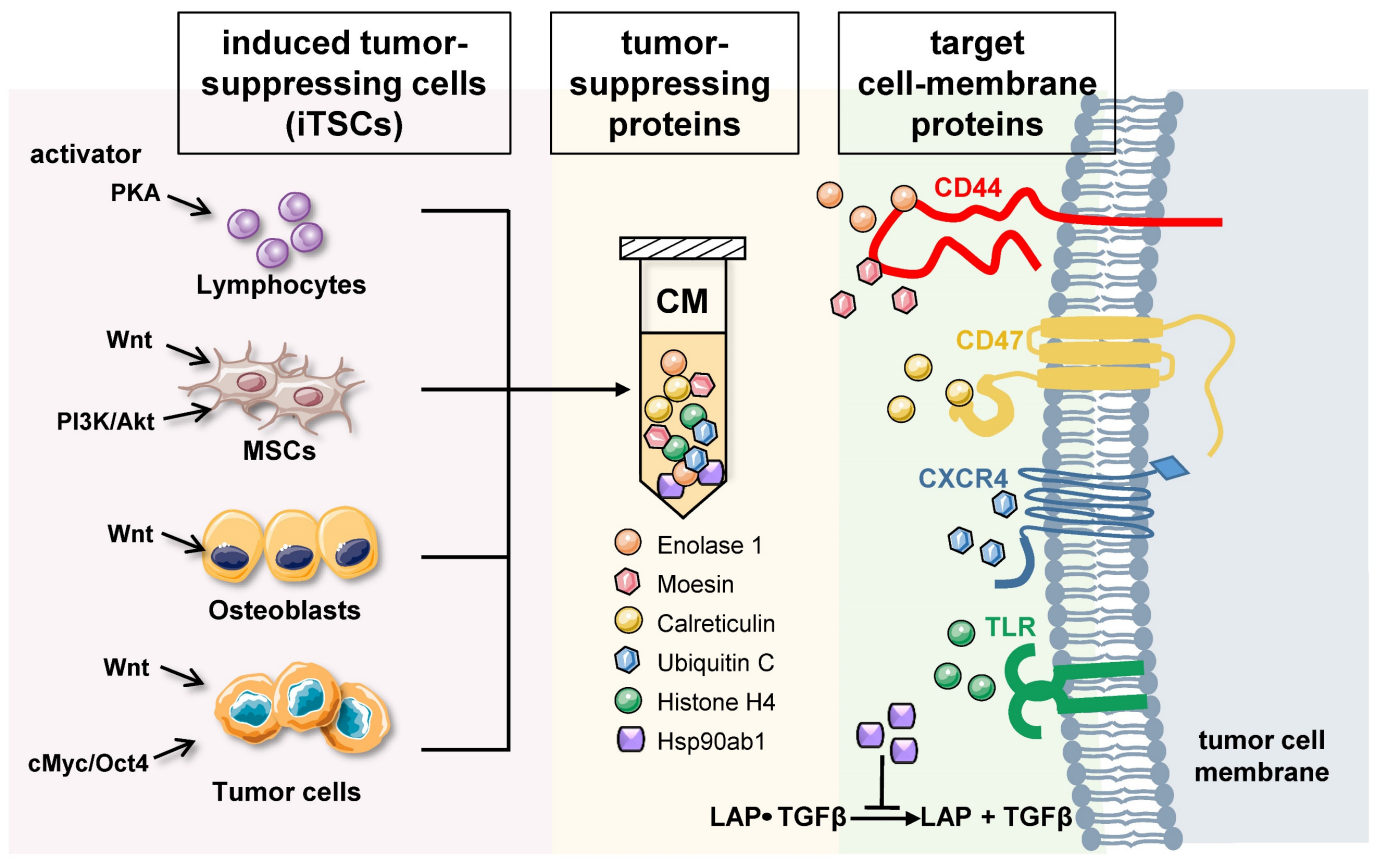
Generation of iTSCs and the proposed mechanism of tumor-suppressive action of their CM. Tumor-suppressing proteins in CM include Enolase 1, Moesin, Calreticulin, Ubiquitin C, Histone H4, and Heat shock protein 90ab1. Enolase 1 and Moesin are enriched in CM, and the interaction with CD44 is involved in their anti-tumor action. Calreticulin acts as an extracellular tumor suppressor by interacting with CD47, while UBC may exert the antitumor effect by its binding to CXCR4. Extracellular histones are known to act as damage-associated molecular pattern proteins by activating TLR4. Furthermore, HSP90ab1 binds to the TGFβ-latent complex and inhibits its generation of mature TGFβ.

**Differential roles of intracellular and extracellular proteins**: Based on whole-genome proteomics analyses followed by *in vitro* cell viability assays, tumor-suppressing proteins, enriched in iTSC-derived CM, have been reported 12, 13, 16. While the current list of CM-enriched tumor-suppressing proteins is limited, approximately 20 proteins have been predicted, and their anti-tumor actions have been validated using recombinant human proteins. Among them, the actions of 6 tumor-suppressing proteins, ENO1, Ubiquitin C (UBC), Moesin (MSN), heat shock protein 90ab1 (HSP90ab1, aka HSP90β), Calreticulin (CALR), and Histone H4 (H4), have been documented with a proposed mechanism (**Figure 1**) 12-14.

**Enolase 1 (ENO1)** - ENO1 is a “moonlighting protein” that functions as a glycolysis enzyme, a plasminogen receptor, and a DNA-binding protein 32. It was a surprise when ENO1 proteins reduced MTT-based viability, EdU-based proliferation, and scratch-based motility of several lines of cancer cells 12, because many lines of evidence support its tumorigenic actions (reviewed in 33). Besides binding plasminogen and participating in the rearrangement of ECM, extracellular ENO1 was shown to interact with CD44, a cell surface adhesion receptor 12, 16. It is proposed that ENO1's anti-tumor action is in part mediated by CD44 which is known to promote cell proliferation in breast cancer cell lines.

**Ubiquitin C (UBC)** - UBC is one of the four ubiquitins in humans, which facilitate the degradation of substrate proteins via ubiquitination. Extracellular UBC presents anti-tumor action to multiple lines of breast cancer cells and pancreatic cancer cells, although the mechanism of its tumor-suppressive capability is not yet elucidated. Because of its role in ubiquitination, UBC interacts with many cell surface proteins. Among them, CXC-motif chemokine receptor 4 (CXCR4) 34, leptin receptor (LEPR) 35, and Hepatitis A virus cellular receptor (HAVCR1) 36 are linked to tumor progression. UBC may also interact with epidermal growth factor receptor (EGFR), whose inactivation by tyrosine kinase inhibitors is effective in improving the survival rates and quality of life of many cancer patients 37. Further studies are needed to examine whether these molecules are involved in UBC-driven tumor suppression.

**Moesin (MSN)** - MSN is one of the three members of the ERM protein family, and as a cytoskeletal adaptor protein, it connects plasma membranes with actin-based cytoskeletons. Existing studies strongly indicate its tumorigenic role. MSN expression by tumor cells is reported to be an unfavorable prognostic biomarker for oral cancer 38. Its high expression is considered a predictor of poor prognosis of breast cancer 39. Also, MSN is a glioma progression marker that induces proliferation and Wnt/β-catenin pathway activation 40. Thus, the anti-tumor action of extracellular MSN, which is mediated by CD44, a transmembrane protein, was unexpected 14. CD44 was first identified as a hyaluronan-binding protein and has been reported both as a tumor suppressor and tumor promoter (reviewed in 41,42). As described in the commentary by Thorne *et al*. 43, CD44 coordinates adhesive and signaling events via its transmembrane and cytoplasmic domains. Both MSN and CD44 function in a context-dependent fashion, and their interactions in the extracellular domain are considered necessary for their anti-tumor action.

**HSP90ab1 (HSP90β)** - HSP90β is a heat shock protein that assists as a chaperone folding of other proteins. In many cancer types including lung cancer, its elevation is linked to metastasis and poor survival 44. It is also reported that targeting HSP90 with chemical inhibitors would degrade these oncogenic proteins, and thus serve as useful anticancer agents 45. It also stabilizes LRP5 and promotes EMT by activating AKT and Wnt/β-catenin signaling 46. However, Suzuki & Kulkarni 47 observed that extracellular HSP90β, secreted from MG63 osteosarcoma cells, binds to TGFβ latent complex and inhibits its activation to generate mature TGFβ. TGFβ is known to exert its protumorigenic function in primary bone tumors by promoting angiogenesis, bone remodeling, and cell migration, and by inhibiting immunosurveillance 48.

**Calreticulin (CALR)** - Like HSP90ab1, CALR is a chaperone protein but its main location is the endoplasmic reticulum. It can be considered a tumor suppressor since it promotes phagocytic uptake of cancer cells when expressed on the cell surface 49. We have shown that extracellular CALR acts as a tumor suppressor by interacting with CD47 50. CD47 is a widely expressed cell membrane receptor that interacts with TSP-1 for angiogenesis, and integrins for cell adhesion and migration, as well as signal-regulatory protein (SIRP) for the inhibition of phagocytosis 51. Various types of cancer express high levels of CD47 to escape from the immune system, and it is a prominent target in cancer therapy 52.

**Histone H4** - Histones are highly conserved intra-nuclear proteins that support the chromatin structure and the regulation of transcription activities. Though they do not serve as tumorigenic factors in the nucleus, extracellular core histones such as H2A, H3, and H4, which are essential components in forming an octamer in chromatin, are reported to be cytotoxic. The intravenous injection of core histones at 75 mg/kg was lethal to mice within 1 h 53. Upon tissue insult such as acute organ injury, extracellular histones are known to act as damage-associated molecular pattern (DAMP) proteins by activating toll-like receptors 2, 4, and 9 (TLR2/TLR4/TLR9) followed by the release of proinflammatory cytokines 54 (reviewed in 55).

The observed role of CM-enriched proteins, which can be either oncogenic or tumor-suppressive, depending on the cellular context, reminds us of the role of the Notch signaling cascade (reviewed in 56). While its aberrant activities are known to initiate and enable the progression of various tumors, the tumor-suppressive role of NOTCH is reported during the development of squamous cell carcinomas 57. It is also reported that NOTCH1 functions as a tumor suppressor in a mouse model of Kras-induced pancreatic ductal adenocarcinoma 58. Taken together, accumulating evidence suggests that cancer cells often employ an individual protein for two contrasting roles. One for their proliferation and migration intracellularly, and the other for the elimination of less-fit neighboring cancer cells extracellularly. Among extracellular tumor-suppressing proteins, some proteins (e.g., HSP90β) are considered secretory proteins, while others (e.g., CALR) are cell-surface proteins. Some other proteins such as histones are not considered secretory proteins. It is of course necessary to understand how these proteins are moved to the extracellular domain by secretory pathways, exocytosis, or cell death.

**Tumor selectivity**: For the selective advantage of super-fit cancer cells, their CM seems fine-tuned to kill less-fit cancer cells but not super-fit cells themselves, or non-cancer cells. The tumor selectivity was analyzed using the reduction in the viability of tumor cells to that of non-tumor cells 13-16, 18. A tumor selectivity larger than one indicates a favorable tumor-selective inhibition, and iTSC CM is reported to kill cancer cells more preferentially than non-cancer cells 13-16, 18. As a potential mechanism for tumor selectivity, we postulate that the levels of target proteins can be elevated in tumor cells rather than non-tumor cells. One such example is the expression of CD47 59. We have shown previously that a tumor-suppressing protein, CALR, inhibited the progression of osteosarcoma cells via interaction with CD47. Consistently, the level of CD47 was elevated in osteosarcoma cells compared to non-tumor cells. Although the mechanism of tumor-selective inhibition is yet to be elucidated, the observed tumor selectivity is a selfish strategy for super-fit cancer cells for eliminating competitors without harming supportive normal cells.

## Application of iTSCs, CM, and protein cocktails

**Figure 2:**
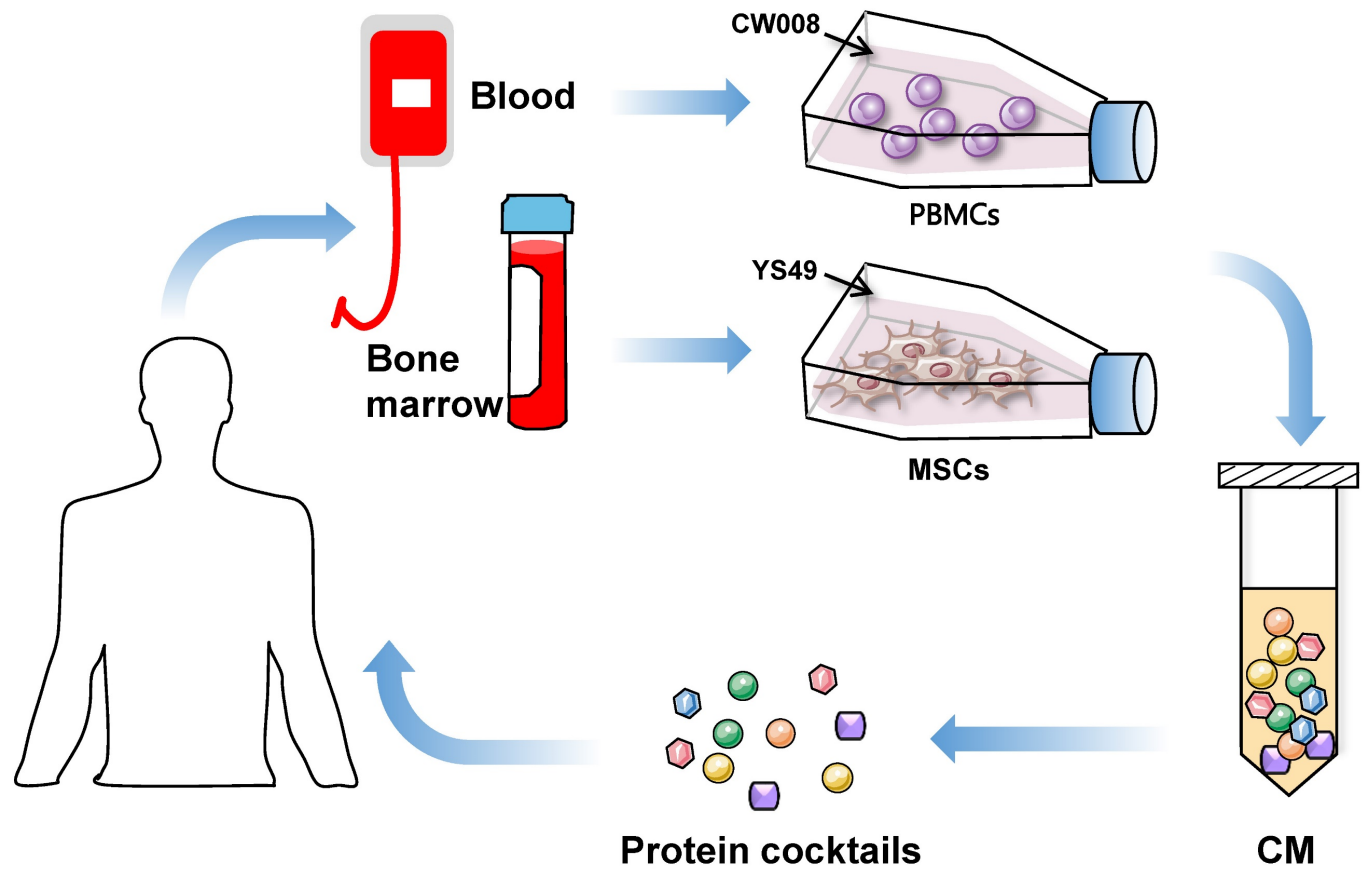
Proposed strategy of the generation of iTSC-derived CM and protein cocktails using patient-derived peripheral blood mononuclear cells (PBMSc) and bone marrow-derived mesenchymal stem cells (MSCs).

The use of iTSC-derived CM and their protein components mimics the cancer cell to eliminate other cancer cells. The dilemmas in agent-based cancer treatments have been the significant side effects during extinction therapy and the development of resistance in adaptive therapy. Evolutionarily, super-fit iTSCs and their CM can be more competitive than conventional chemotherapeutic agents, since they are designed and produced to win against competitors by artificial bench-side selection. To generate potent iTSCs and their CM, it is necessary to develop biological and chemical agents not for killing cancer cells but for converting cancer and non-cancer cells into super-fit cells (**Figure 2**).

**ITSCs**: Because of their availability and clinical applicability, MSCs and PBMCs are good choices for generating iTSCs. MSCs are used mainly in regenerative medicine, while T cells in PBMCs are used for chimeric antigen receptor (CAR)-T immunotherapy. This immunotherapy employs T cells with the transfection of chimeric antigen receptors, and its therapeutic ability has been shown in many cancer types including advanced leukemia and lymphoma 60. Of note, any mutations and epigenetic modifications in host cells need to be examined when autologous cells from cancer patients are utilized. Patient-derived iTSCs may generate proteins and peptides that can be differentially processed 61. Of note, MSCs are immune privileged, and allogenic MSCs will not elicit inflammatory responses, mainly due to their lack of class-II major histocompatibility complex and costimulatory molecules 62. A potential concern with MSCs is limited survival rates as well as migration and homing ability 63, 64. To enhance their therapeutic efficacy, varying pre-activation strategies are considered, including genetic modification, chemical treatment, and mechanoelectrical stimulations (reviewed by 65). Besides gene overexpression and chemical activation of tumorigenic pathways, it is of interest to test whether electromechanical stimulations may enhance the anti-tumor capability of iTSC CM. To avoid the risk of *in vivo* proliferation and differentiation, enucleated MSCs, named cargocytes, have recently been developed 66.

The use of PBMCs is another choice as host cells for iTSC generation. PBMCs contain lymphocytes (T cells, B cells, and NK cells) in the range of 70-90%, monocytes from 10 to 20%, and other cells such as dendritic cells 67. T lymphocytes are employed for chimeric antigen receptor (CAR) T-cell immunotherapy, in which T cells are genetically engineered to produce an artificial T cell receptor that can bind an antigen specific to a particular type of cancer 68, 69. PBMCs, collected from cancer patients and healthy individuals, were successfully converted to iTSCs, and the anti-tumor capability of their CM was verified by *ex vivo* tissue models as well as mouse models 50. Besides MSCs and PBMCs, iTSCs can be generated from many other host cells including varying cancer cells. It is yet to be tested whether iTSCs derived from patients' cancer cells have an advantage over non-cancer cell-derived iTSCs.

**CM**: CM is a mixture of various biomolecules including microRNAs, circulating tumor cell DNAs, etc., whereas the prime components summarized in this review are proteins. The advantage of CM is its integrity and robustness as a single therapeutic agent with thousands of components. To attack super-fit cancer cells, CM is well equipped to face many branches of an evolutionary clade with an array of diverse tumor-suppressing proteins. Procedurally, CM should be prepared in an artificial culture medium without animal serum. It is important to define the base medium since the culture conditions significantly affect the proteomes in CM 70. *In vitro* characterization revealed that the core tumor-suppressing components are proteins above 3 KD since the treatments such as nuclease digestion, filtering with 3 KD cutoff, and ultracentrifugation for exosome removal did not significantly alter the anti-tumor ability. The protein concentration of CM is adjustable by buffer exchange, and IC_50_ can be obtained for the standardized CM (e.g., CM adjusted at 1 mg/mL protein concentration).

**Protein cocktails**: Alternatively, it may be possible to select potent tumor-suppressing proteins to construct a protein cocktail that may be customized for individual patients. Antibody-based cancer therapy is rapidly evolving for the regulation of target proteins, engagement of cytotoxic T cells, and delivery of cytotoxic payloads (antibody-based cancer therapy oncogene) 71. By contrast, the administration of recombinant human proteins such as tumor necrosis factor-related apoptosis-inducing ligand (TRAIL) presents poor pharmacokinetics and weak potencies because of its short half-life 72. Depending on the stability and potency of individual tumor-suppressing proteins, an advanced delivery system to improve half-life, targeting efficiency, bioavailability, and bioactivity needs to be developed. An example of an advanced formulation capable of high loading of proteins or peptides has recently been reported using flash nanoprecipitation 73. Such systems can encapsulate proteins and peptides and also provide targeting ability towards a desired tumor/bone niche.

## Linkage to Warburg effect and induced pluripotent cells (iPSCs)

**Warburg effect**: Besides a double-edged role of a majority of atypical tumor-suppressing proteins, an interesting linkage to glycolysis can be pointed out. In the 1920s, Otto Warburg observed that cancer cells preferentially generate energy by glycolysis even in the presence of oxygen. While this Warburg effect is a reprogramming of cell metabolism, it is still controversial how the Warburg effect benefits cancer cells 74. Aerobic glycolysis may accumulate lactate and acidify the extracellular domain 75. The former simulates the sustained proliferation of cancer cells and suppressed anti-tumor immunity, while the latter accelerates malignant progression and drives resistance to conventional therapies. Notably, many glycolytic enzymes are reported present in the serum of breast cancer patients, including aldolase A (ALDOA), ENO1, and glyceraldehyde-3-phosphate dehydrogenase (GAPDH) 76. Furthermore, among the list of 56 proteins enriched in a Wnt-activated iTSC-derived conditioned medium 12, eight glycolytic enzymes were included, such as ALDOA, ENO1, PGAM1 (phosphoglycerate mutase 1), LDHA (lactate dehydrogenase A), PKM (pyruvate kinase M), TPI1 (triosephosphate isomerase 1), GAPDH, and PGK1 (phosphoglycerate kinase 1). While the extracellular tumor-linked roles of PGAM1, LDHA, PKM, TPI1, and PGK1 have not been tested, the anti-tumor actions of ALDOA 13, 18, ENO1 16, 18, and GAPDH 20 were reported. Collectively, an intriguing question is whether the Warburg effect may contribute to generating a double-sided environment, not only favoring tumor growth by lactate accumulation and acidification but also utilizing a group of glycolytic enzymes as extracellular tumor-suppressing proteins.

**Linkage to iPSCs**: The Warburg effect is associated with metabolic reprogramming, while iPSCs are linked to cell fate reprogramming. The reprogramming efficiency of iPSCs can be elevated by reducing apoptotic and senescent cells during the process of iPSC transformation. Cell senescence is a state of stable, terminal cell cycle arrest, and a growing number of studies have convincingly demonstrated the double-edged role of the secretomes of two types of senescent cells 77. In the process of iPSC generation, senescent cells can be induced by activating oncogenes. Interestingly, oncogene-induced senescent cells mediate tumor suppression in a cell-extrinsic manner 78. By contrast, the other type of senescent cells, which is induced by cellular and therapeutic stresses, can secrete senescence-associated factors that mediate tumor progression 79. Thus, oncogene-activated cell-derived secretomes can be tumor suppressive, while chemotherapeutic agent-treated cell-derived secretomes may act as tumor stimulators. Collectively, it is recommended to investigate a potential linkage between oncogene-induced cell-derived secretomes in iPSC generation and iTSC-derived secretomes.

## Future Perspectives

As cancer cells evolve and enhance their fitness, their CM also evolves. The therapeutic task is “how to respond to a wide spectrum of aggressive cancer cells in patients by generating a broad range of super-fit iTSCs on the bench side”. The future therapeutic strategy should consider the development of activators rather than inhibitors to generate super competitors, and treatments should be directed to advanced and metastasized cancer of many types and subtypes. Here are 5 items further studies should investigate.

- Variations in CM-enriched proteins, depending on host iTSCs: iTSCs can be developed from a wide spectrum of tumor cells and non-tumor cells. Tumor-suppressive CM can also be generated from tissues including freshly isolated carcinomas and sarcomas. Since different types of cells synthesize different proteins, it is reasonable to assume that CM-enriched proteins differ depending on host iTSCs. A question is “whether a group of tumor-suppressing proteins such as histones is common among CMs”. Also, since protein interactions heavily rely on modifications due to mutations, rearrangements, epigenetic changes, etc., we need to evaluate the role of protein isoforms and alterations.
- Dependence of CM's efficacy on cancer types and subtypes: Depending on the pathway to be activated, the efficacy of CM in cancer types and subtypes should differ. For instance, a question is whether Wnt-activated iTSC CM is most effective in treating Wnt-dysregulated cancer cells.
- Possibility of CM generation by deleting tumor-suppressing pathways: Tumor cells may enhance their proliferation not only by activating tumorigenic signaling but also by inactivating anti-tumorigenic signaling. Besides activating PKA signaling in PBMCs, we have successfully developed iTSCs by inhibiting AMPK signaling (80, 81). The reciprocal procedure of generating iTSCs may widen the therapeutic possibilities with iTSCs.
- Contribution of non-protein factors to CM's tumor-suppressing capabilities: Besides tumor-suppressing proteins, other molecules such as peptides, nucleic acids, lipids, and varying metabolites can be involved in CM's tumor-suppressing capabilities, although the capabilities are not significantly altered by nuclease digestion, or the removal of small molecules (3 KD cutoff) and exosomes 15, 16.
- Compatibility of CM with existing chemotherapeutic drugs and immunotherapy: Finally, it is desirable if CM can contribute to lowering the dose of chemotherapeutic drugs and lessening their side effects. Furthermore, the effect of iTSC CM on the immune system should be studied. It is reported that CM downregulates programmed cell death ligand-1, PDL1 12, 14, 16

Besides protein cocktails, a cocktail of CMs, or a mixture of multiple CMs, can be tested to cope with a wide spectrum of cancer types. Whether the treatment may enter the adaptive, taming phase after the failure of extinction depends on the ability to generate stronger super-fit cells on the bench side than natural selection-driven patients' cancer cells.

In summary, accumulating evidence demonstrates that the use of inhibitory agents such as cytotoxic chemotherapeutic drugs tends to promote the generation of tumor-promoting proteomes, while the use of cell-proliferative and tumorigenic agents such as pharmacological Wnt activator induces the production of tumor-suppressive proteomes. The insubordinate response of tumor cells results from the evolutionary legacy for them to survive. We observed three paradoxical maxims in the behavior of tumor cells with their proteomes. First, proliferating tumor cells remove neighboring tumor cells by secreting tumor-suppressing proteins. Second, some of the secreted tumor-suppressing proteins are oncogenic inside the cell. Third, they preferentially kill tumor cells rather than non-tumor cells. As shown in recent reports, by activating tumorigenic signaling, iTSCs can be generated, and their CM can be employed to suppress tumor progression in preclinical studies. iTSC CM was shown to be enriched with atypical tumor suppressors such as ENO1, UBC, MSN, HSP90ab1, CALR, and histone H4. Further research is necessary to warrant the efficient and safe application of iTSCs, their CM, and cocktails with CM-enriched proteins, in extinction therapy and adaptive therapy with and without existing therapeutic agents. Repurposing not only drugs but also proteins in a combinatorial way may hold considerable promise in precision oncology and personalized medicine 82.
